# A Puzzle Unsolved: Failure to Observe Different Effects of God and Religion Primes on Intergroup Attitudes

**DOI:** 10.1371/journal.pone.0147178

**Published:** 2016-01-26

**Authors:** Jonathan E. Ramsay, Eddie M. W. Tong, Joyce S. Pang, Avijit Chowdhury

**Affiliations:** 1 UniSIM College, SIM University, Singapore, Singapore; 2 Department of Psychology, National University of Singapore, Singapore, Singapore; 3 Division of Psychology, Nanyang Technological University, Singapore, Singapore; 4 Centre for Applied Research, SIM University, Singapore, Singapore; University of Akron, UNITED STATES

## Abstract

Religious priming has been found to have both positive and negative consequences, and recent research suggests that the activation of God-related and community-related religious cognitions may cause outgroup prosociality and outgroup derogation respectively. The present research sought to examine whether reminders of God and religion have different effects on attitudes towards ingroup and outgroup members. Over two studies, little evidence was found for different effects of these two types of religious primes. In study 1, individuals primed with the words “religion”, “God” and a neutral control word evaluated both ingroup and outgroup members similarly, although a marginal tendency towards more negative evaluations of outgroup members by females exposed to religion primes was observed. In study 2, no significant differences in attitudes towards an outgroup member were observed between the God, religion, and neutral priming conditions. Furthermore, the gender effect observed in study 1 did not replicate in this second study. Possible explanations for these null effects are discussed.

## Introduction

All major world religions claim jurisdiction over morality; professing that their creed yields righteous behavior that is pleasing to their God, Gods, or conception of The Divine. Christians believe that accepting Jesus Christ gives rise to “fruit of the Spirit”, which encompasses such moral fundamentals as “love, joy, peace, forbearance, kindness, goodness, faithfulness, gentleness, and self-control” [1]. Similarly, the Qur’an teaches that “those who believe, and do deeds of righteousness, and establish regular prayers and regular charity, will have their reward with their Lord” [2]. Similar passages can be found in the holy books of all major faiths, such as the Buddhist Tipitaka [3], the Hindu Vedas, [4], and the Jewish Talmud [5]. Indeed, religion is seen by many believers to be a prerequisite for moral behavior. A 2002 poll by the Pew Research Center found that 60% of Americans believe that a religious upbringing gives rise to a morally righteous life, while almost half claim that belief in God is a prerequisite for morality. Consequently, atheists are viewed by believers as being prone to immorality precisely because their actions are not constrained by divine moral laws [6], while religious individuals ascribe greater morality to fellow believers [7].

This morality is reflected in the behavior of many religious individuals, who frequently display altruistic behavior on both a global (e.g. [8]) and local (e.g. [9]) scale. Nonetheless, it is also undeniable that violence and discrimination are frequently committed in the name of religion [10, 11]. Religious groups are often complicit in the marginalization of minorities (e.g. [12]), and many deadly conflicts are fought between opposing religious groups (e.g. [13, 14]). This coexistence of religious morality and immorality represents a central paradox of religious belief: the capacity of religion to promote both tolerance and prejudice.

Findings in the psychological literature reflect this apparent divergence in function. Studies of trait religiosity have found that religious individuals behave more prosocially across a range of domains, such as charitable giving [15, 16], volunteering [16, 17], and cooperation in laboratory tasks [18], while well-documented relationships also exist between individual differences in religiosity and various forms of prejudice, such as anti-homosexual prejudice [19], racial prejudice [20], and ethnic prejudice [21]. Recently, empirical investigations have moved beyond correlational designs in order to identify causal relationships between religion and prosociality/antisociality. Priming religion has been found to increase cooperation in economic games [22, 23], promote socially desirable responding [24], increase interest in charitable involvement [25], and reduce cheating in anonymous situations [26], while also increasing prejudice against racial minorities [27] and homosexuals [28]. Such primes have also been shown to increase both submissiveness [29] and conformity [30], two qualities that may sustain prejudicial views that are proscribed by legitimate authorities.

In seeking to understand these opposing forces, many psychologists have invoked classical theories of intergroup relations such as social identity theory [31] and self-categorization theory [32]. As exclusive social groups that are central to a believer’s self-concept [33], religious belonging should promote intergroup differentiation, leading to ingroup favoritism and outgroup derogation. Consistent with this account, religious prosociality is often limited by the proximity and familiarity of the target [34]. Saroglou, Pichon, Trompette, Verschueren, and Dernelle [35] found that religiosity predicted helping relatives and acquaintances but not unknown others, while Johnson, Rowatt, and LaBouff [36] observed that religious traits and primes enhanced attitudes towards ingroup members vis-à-vis attitudes towards outgroup members. Pichon and Saroglou [37] found that religious primes increased willingness to help a homeless person of the same nationality but not an illegal immigrant. This has been termed *parochial altruism*: the tendency for individuals to favor ingroup members over outgroup members when helping at a personal cost [38]. Parochial altruism is also consistent with evolutionary accounts of the origin of religion, which propose that shared religious beliefs arose because of their ability to “bind people together into cooperative communities organized around deities” ([39] p. 140). It has been argued (e.g. [23, 40]) that religious communities, in which a supreme being has the capacity to reward morality and punish immorality, should cooperate more successfully than non-religious communities, and therefore be more likely to proliferate and to survive.

Nonetheless, instances of prosociality directed toward outgroup members suggest the situation is more complex. The enhanced philanthropy exhibited by religious individuals extends to secular charities as well as religious ones [15, 17], while spirituality has been shown to relate to universalism [41] and to predict willingness to help individuals of uncertain religious affiliation [30]. Furthermore, individuals high in quest religiosity do not discriminate between value-violating outgroup members and value-upholding ingroup members when choosing whether to offer help [42]. Given that religious prosociality occasionally crosses intergroup boundaries, straightforward social identity explanations involving intergroup differentiation do not seem entirely adequate.

Preston and colleagues [43, 44] have attempted to address this issue by formulating a dual-process model of religious prosociality. They have argued that positive interpersonal behaviors can be promoted by two different yet equally fundamental aspects of religion: (a) the belief in a moralizing deity, and (b) the notion of religion as a community bonded by a shared worldview. They argue that the former should promote benevolence towards all, given that the sovereignty of their God or Gods applies to everyone, while more earthly concerns with their religious community should elicit parochial altruism. They also suggest that some of the conflicting results of religious priming studies (e.g. [23, 27]) may be due to conflation of these two distinct aspects of religion.

This distinction between the supernatural and institutional effects of religious cognition on interpersonal behavior has been examined in a recent study. Preston and Ritter [44] investigated the effects of supernatural versus religious institutional priming on helping and cooperating with ingroup and outgroup members. They found that individuals primed with the word “religion” displayed enhanced charitable giving and cooperation in a laboratory task when the target of their behavior was presumed to be a fellow ingroup member. Conversely, individuals primed with the word “God” displayed greater generosity and cooperation when the target was presumed to be an outgroup member. Together, these results support their dual-process model, suggesting that activation of supernatural religious cognitions promotes altruism toward outgroup members, possibly due to concerns regarding supernatural monitoring, while activation of religious institutional cognitions activates concerns for the protection of the ingroup, resulting in parochial altruism.

These intriguing findings beg an important question. It is currently unclear whether Preston and Ritter’s [44] findings can be extended to the attitudinal domain, given that enhanced prosociality was investigated using two behavioral measures. While investigating effects on observable behavior is of paramount importance, it is also important to understand effects on affect and cognition, the other two components in the triarchic model of attitudes [45]. As such, it is important to investigate the effects of supernatural and religious institutional primes on stereotypic beliefs regarding ingroup or outgroup members, such as appraisals of their warmth or ability [46].

### The Present Research

The present research sought to extend the research described above by providing the first empirical investigation of the effects of God (supernatural) and religion (institutional) priming on attitudes—both positive and negative—towards ingroup and outgroup members. Specifically, we sought to examine how God and religion primes affect intergroup attitudinal judgments.

Following the argument of Preston and Ritter [44], we anticipated divergent effects for these two types of religious prime. For individuals exposed to religious institutional primes, we anticipated an increase in parochial altruism, manifesting predominantly as outgroup derogation rather than ingroup favoritism. While Johnson, Rowatt, and LaBouff [36] reported that religious priming (in this case both supernatural and religious institutional primes) resulted in increases in both ingroup favoritism and outgroup derogation, their results were obtained using difference scores reflecting disparities in attitudes. Usage of difference scores causes interpretative difficulties [47], and it is hard to definitively conclude whether ingroup favoritism, outgroup derogation, or a combination of the two phenomena drove these effects. Ramsay, Pang, Shen, and Rowatt [28] found that mixed supernatural and religious institutional primes yielded more negative attitudes towards an outgroup in both Christians and Buddhists, suggesting that outgroup derogation is an important consequence of exposure to certain kinds of religious primes. However, the use of a mixture of both supernatural and religious institutional primes together in these two studies obfuscate the results further as it is unclear whether it was supernatural or religious institutional primes that was driving the effects. The present research sought to clarify these previous findings by examining the effects of God and religion primes separately and by avoiding the use of difference scores.

For individuals primed with supernatural religious primes, we expected more positive attitudes to all individuals, irrespective of group affiliation. Given the pan-religious applicability of the Golden Rule [48], it seems theoretically intuitive that God primes should enhance attitudes towards members of all groups, rather than causing individuals to favor members of certain groups over others. Although Preston and Ritter [44] found that supernatural primes enhanced bias in favor of outgroups, the same authors ([43] p. 585) stated that: “The mental representation of God as benevolent establishes a standard of universal prosociality, untainted by prejudices or preferences…As a result, thoughts of God may promote good will toward all others, not just the ingroup.” As such, we anticipated that individuals primed with God primes would exhibit significantly more positive attitudes towards ingroup members as well as outgroup members, compared to individuals primed with either religion or neutral primes.

Given the above, the following hypotheses were made:

- H1a: Attitudes toward an outgroup member would be significantly more negative in the religion prime condition than in the neutral or God prime conditions.
- H1b: In the religion prime condition, attitudes toward an outgroup member would be significantly more negative than attitudes toward an ingroup member.
- H2: Attitudes toward both the ingroup and outgroup member would be significantly more positive in the God prime condition than in either the neutral or religion prime conditions.

## Study 1

In study 1 we chose to test each of these hypotheses in a laboratory-based study utilizing supraliminal priming methods. The study adopted a 3 × 2 between-subjects factorial design, with prime content (God vs. religion vs. neutral control) and evaluation target (ingroup member vs. outgroup member) as independent variables. The experiment was presented to participants as an investigation of “personality, cognitive ability, and critical skills” in order to disguise the true intent and to guard against hypothesis awareness. Prior to attending the single laboratory session, all participants completed a short online questionnaire that assessed their demographic characteristics and several additional variables that are not the focus of the present analysis.

### Method

#### Participants

In total, 232 students from Nanyang Technological University in Singapore participated in the study. The sample was 65.9% female with a mean age of 20.82 years (*SD* = 1.65). The majority of the sample was ethnically Chinese (79.7%), with Malays (7.8%), ethnic Indians (5.2%), individuals of Western European descent (1.3%) and other ethnic groups (6.0%) making up the remainder of the sample. All ethnic categorizations were made by the participants themselves, and were required only to ensure that different groups were adequately represented. The sample was heterogeneous in terms of religious identification. Nearly one third of the sample identified as either Buddhist or Taoist (33.2%), with Christian/Catholic (29.8%), Muslim (8.6%), and Hindu (3.4%) the other religious groups represented. Exactly one quarter of the participants identified as “free thinkers”, which is a commonly used term in Singapore that encompasses atheists and agnostics, as well as those who have no religious views or affiliation.

This research was approved by the Nanyang Technological University Institutional Review Board prior to the commencement of data collection. All participants provided informed written consent before completing the pre-laboratory questionnaire, and were fully debriefed after the experiment was complete. Partial course credit was awarded to all participants as recompense for their time.

#### Design and procedure

**Fig 1:**
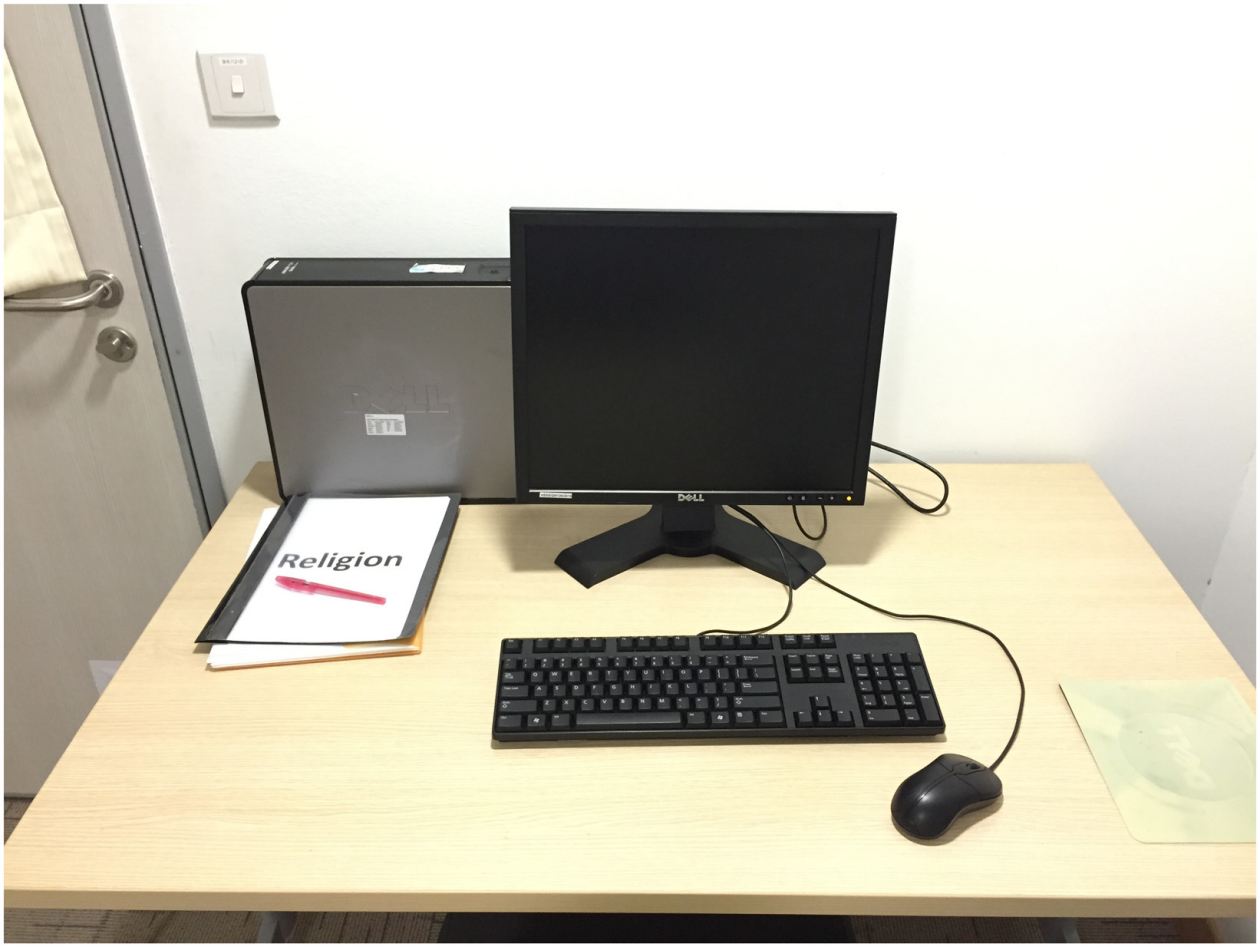
A depiction of the administration setting, including a computer (for task completion) and priming materials.

All participants were randomly assigned to one of the six experimental conditions prior to their arrival at the laboratory. Experimental sessions were conducted with groups of no more than four participants, with participants first being briefed on the experimental procedure as a group, before being directed to individual cubicles where each participant completed the main experimental tasks in isolation. A photograph of the individual administration setting can be found in Fig 1. This procedure was essential for ensuring the effectiveness of the priming procedure, as will be detailed below.

After being seated in their individual cubicles, participants first completed an essay evaluation task that provided a measure of attitudes towards either ingroup or outgroup members, depending on their group allocation. Participants subsequently completed several additional questionnaires pertaining to such constructs are psychological essentialism, self-accessibility, and impression management (the results of which are not examined in the present analysis), as well as a suspicion check and a distractor task. Once the participants had finished all the tasks, they were thanked and told they could leave. In order to guard against communication of the experimental hypothesis among acquainted participants, all participants were debriefed via email *en masse* once data collection had been completed.

#### Materials

God, Religion, and Neutral Primes: Target constructs were primed using a supraliminal priming procedure adapted from Chan, Tong, and Tan [49], in which ostensibly unrelated priming materials were placed in clear view of the participants. In each individual administration cubicle, participants were seated at a personal computer that was used to administer the various tasks and measures. The primes, disguised as piles of study materials, were positioned immediately to the left of the computer monitor at the corner of the desk (see Fig 1). At the top of each pile was a folder with a cover that varied according to the participant's group allocation. In the God prime condition, the word “God” was written on the folder's cover page in a large (132 pt.), sans-serif typeface, whereas in the religion and neutral conditions the word “God” was replaced with the words “Religion” and “Neutral” respectively. In each case, a pen (that did not obscure the prime word) was placed on top of the folder, while several periodicals and textbooks were placed underneath. This was in order to disguise the prime as a pile of discarded study materials accidentally left over from a previous usage of the room.

When leading participants into the room, the experimenters made sure not to draw attention to the priming materials. The experimenters did not mention or look at the primes when giving instructions to the participants, and none of the participants mentioned or asked questions regarding the priming materials. After being seated, participants were left unattended for one minute to ensure that they saw the priming materials, after which they would begin the essay evaluation task. In the suspicion check, participants were asked to speculate on the experiment's true purpose by choosing one of seven options: “attitudes and prejudice”, “logic and reasoning”, “language fluency”, “perceptual ability”, “critical thinking”, or “memory”. The majority (51.5%) chose “perceptual ability”, while only 7% chose the first option. Of those participants who believed that the study investigated attitudes and prejudice, none mentioned the religious primes or successfully identified the manipulation in their answers to an open-ended follow-up to the first suspicion check.

Measure of Implicit Attitudes: Attitudes towards ingroup and outgroup members were assessed using an essay evaluation task, in which participants were asked to critically evaluate an essay supposedly written by either an ingroup or outgroup member. Evaluations of written material have been used to implicitly assess intergroup attitudes in previous research (e.g. [50, 51]), and a similar technique was adopted in the present study. Participants were asked to read a 460-word essay entitled “Why Should You Complain About Bad Service?” in which the author argued that complaining is necessary if the standard of customer service in Singapore is to be improved. According to the cover story, an undergraduate from a local university wrote the essay as part of an essay competition. Participants were told that the student author was given the essay title and asked to be both as persuasive and as concise as possible when arguing in favor of the allocated position. In reality, the essay was chosen from a large online repository of sample undergraduate essays, with key details modified in order to fit the local Singaporean context. The essay was chosen because of its appropriate length, and also because it displayed reasonably good spelling, grammar, and rhetorical ability without seeming too polished, which may have led participants’ to doubt its authenticity.

In order to ensure that the sample essays appeared to be legitimate, text entry boxes for the author’s name, university identification number, and signature were included, with the contents blacked-out in order to give the impression that his or her identity was being purposely concealed by the experimenter. The only identifying information that remained unobscured was the contestant’s university, which was designated as either Nanyang Technological University (NTU) or the National University of Singapore (NUS), in order to identify the author as either an ingroup or outgroup member, respectively. NUS and NTU are the two largest universities in Singapore, together accounting for over 85% of the nation’s undergraduate population [52, 53, 54], and a rivalry exists between students of the two institutions. The affiliation of the fictional student was further emphasized by the presence of a matching university logo in the letterhead at the top of the essay sheet. Reproductions of the essays can be found in the S1 Appendix.

After being seated alone in their individual cubicles for one minute, participants were invited to click a button on the screen to begin. They were subsequently provided with instructions for the essay evaluation task, and then invited to turn over a sheet of A4 paper positioned on the desk directly in front of them. The essay and partially obscured identifying information described above were printed on the reverse. Participants were subsequently asked to rate the essay’s quality on six individual dimensions: “persuasiveness of argument”, “quality of supporting examples”, “flow and cohesion”, “readability and coherence”, “spelling and grammar”, and “vocabulary and verbal ability”. Participants were also required to answer two global evaluation questions: “How much did you enjoy reading this essay?” and “Overall, how would you rate the quality of this essay?”. All answers were provided on 11-point Likert scales anchored at *0 = terrible* and *10 = excellent*, with the exception of the enjoyment question, which was anchored at *0 = not at all* and *10 = very much*. Scores on the eight evaluative indices were found to be highly correlated, with *r* values ranging from .308 to .799, all of which were significant at the *p* = .01 level. As such, the eight variables were collapsed into a single variable representing an overall appraisal of the essay’s quality. This was to be the sole dependent variable examined in the subsequent analyses.

### Results

A two-way between subjects ANOVA was conducted to compare the effects of priming condition and group membership on essay evaluation scores. Priming condition comprised three levels (God prime, religion prime, and neutral prime) and target group membership comprised two levels (ingroup and outgroup). There was no main effect of priming condition on essay evaluation, *F*(2, 226) = 1.22, *p* = .23, nor was there any main effect of group membership, *F*(1, 226) = .558, *p* = .46. Importantly, there was also no interaction effect between priming condition and group membership, *F*(2, 226) = .586, *p* = .56. Together, these results did not offer any support for the hypothesized effects of God and religion primes on attitudes towards ingroup and outgroup members.

However, given that significant gender differences in religiosity have frequently been observed [55], additional analyses were conducted in order to test for possible gender effects that may have been obscured in the previous ANOVA. To this end, a three-way ANOVA was conducted in order to investigate whether participants’ gender moderated the effect of either prime or group membership on essay evaluation. The results of this analysis revealed no significant main effects, but the two-way interaction between sex and target was significant, *F*(1, 220) = 6.23, *p* = .01, ηp² = .03, while the two-way interaction between sex and prime was marginally significant, *F*(2, 220) = 2.62, *p* = .08, ηp² = .02. Other interaction effects were not significant: prime × target, *F*(2, 220) = .57, *p* = .57; prime × target × sex, *F*(2, 220) = .69, *p* = .50.

In order to further examine these two-way interactions, post-hoc simple main effects tests were conducted. Descriptive statistics calculated separately for the two sexes can be found in Table 1. None of the simple effects in the analysis of the male participants gave rise to significant results. Analysis of the female data however, revealed several significant differences. There was a simple main effect of target group on essay evaluation scores for the female subsample, *F*(1,220) = 4.62, *p* = .03, ηp² = .02. Pairwise comparison showed that females rated the ingroup essays more positively compared to outgroup essays (*p* = .03). There was also a simple main effect of priming on the outgroup essay evaluation in the female subsample, *F*(2,220) = 3.18, *p* = .04, ηp² = .03. Pairwise comparison showed that Religion priming led to significantly more negative evaluation of the outgroup essays when compared to the God priming condition (*p* = .02). However, outgroup essay evaluations were only marginally more negative in the Religion priming condition than the neutral priming condition (*p* = .06). As such, hypothesis H1a was only partially supported in the female subsample. The results also showed a simple main effect of target group for females in the Religion primed condition, *F*(1,220) = 6.84, *p* = .01, ηp² = .03. Pairwise comparison showed that in the Religion prime condition females rated the outgroup essays significantly more negatively compared to the ingroup essays (*p* = .01), meaning that hypothesis H1b was supported in the female subsample. Finally, there was a marginally significant simple main effect of priming on essay evaluation for the female subsample, *F*(2,220) = 2.72, *p* = .07, ηp² = .02. After combining the ingroup and outgroup conditions, pairwise comparisons indicated that females generally evaluated essays significantly more positively in the God prime condition compared to the Religion prime condition (*p* = .02), but not the neutral condition (*p* = .25). These results offer only limited support for hypothesis H2 in the female subsample.

**Table 1:** Descriptive statistics for essay evaluations by priming condition, target condition, and gender.

Priming Condition	Target Condition	Full Sample					Female Sample					Male Sample				
		*M*	95% CI		*SD*	*n*	*M*	95% CI		*SD*	*n*	*M*	95% CI		*SD*	*n*
God	In-Group	6.35	5.88	6.81	1.45	37	6.59	6.08	7.12	1.46	27	5.68	4.70	6.65	1.28	10
	Out-Group	6.25	5.80	6.70	1.40	40	6.21	5.72	6.70	1.44	30	6.36	5.39	7.34	1.37	10
Religion	In-Group	6.22	5.78	6.66	1.39	42	6.29	5.80	6.78	1.25	30	6.05	5.16	6.94	1.76	12
	Out-Group	5.81	5.34	6.27	1.51	38	5.23	4.65	5.82	1.39	21	6.51	5.77	7.26	1.38	17
Neutral	In-Group	6.31	5.83	6.79	1.61	36	6.11	5.52	6.69	1.50	21	6.59	5.80	7.39	1.78	15
	Out-Group	6.39	5.93	6.85	1.32	39	6.05	5.50	6.59	1.26	24	6.94	6.15	7.74	1.55	15

It should be noted that the pairwise comparisons described in the preceding paragraph were conducted without correcting for multiple comparisons. Bonferroni-correction would give a *p* cut-off value of .017, under which circumstances only hypothesis H1b would have been supported. While there are grounds to be cautious in applying overly conservative alpha corrections on a small number of comparisons, it is important to draw the reader’s attention to this issue, especially given the non-significant results in the full-sample ANOVA.

Finally, to see if the effect of religious priming was stronger in the religious members of our sample, we conducted the 3-way ANOVA after excluding the data from participants who identified as free thinkers or atheists. The results were identical to the primary findings with no significant effect of priming, *F*(2,162) = .68, *p* = .51 or two-way interaction between priming x target group, *F*(2,162) = .67, *p* = 0.50. The two way interaction between gender x target group replicated, *F*(1,162) = 7.05, *p* = .01 and the gender x priming interaction reached statistical significance at alpha = .05 level, *F*(2,162) = 3.24, *p* = 0.04. All other effects were non-significant.

### Discussion

In study 1 we investigated the differential effects of supernatural and religious institutional priming on implicit attitudes towards ingroup and outgroup members, as measured using an essay evaluation task. Overall, the results did not provide convincing support for any of our stated hypotheses. Specifically, no significant differences in essay evaluation scores were found among the three priming conditions, nor was there a difference between ingroup and outgroup evaluations. Critically, there was also no interaction effect between priming condition and group membership on essay evaluation. In absence of a significant interaction, there was no justification for performing the pairwise comparisons corresponding to each of the three hypotheses in the full sample, and so no support was garnered for any of the experimental hypotheses. However, when participant gender was taken into consideration, data for the female subset of the participant pool did partially support several of our hypothesis. Following the observation of significant sex × target and sex × prime interaction effects, pairwise comparisons in the female-only subsample offered some support for H1a and H1b, but not for H2. Even these results come with several important caveats. Firstly, application of Bonferroni correction would have rendered the pairwise comparison associated with H1a non-significant. Secondly, the omnibus three-way ANOVA between prime, target, and gender was non-significant.

One explanation for such findings may be that the intergroup distinction adopted in study 1 may have been too subtle and benign, and therefore lacked sufficient saliency needed to evoke processes of intergroup differentiation in participants. It is possible that the university rivalry, while important in certain competitive contexts (e.g., sports), may not be important to all students. This possibility is supported by the fact that no significant main effect of target group membership on essay evaluation observed in study 1. Another possible explanation for our null findings in study 1 related to the operationalization of our dependent variable. The measure of implicit attitudes focused on a single dimension of interpersonal judgment: competence, as manifested in appraisals of essay writing skill. While the stereotype content model [46] does suggest that judgments of competence are an important component of the stereotypes ingroup members form about members of outgroups, it is possible that adoption of a more global attitudinal measure may have led to the detection of significant prime-induced differences. These concerns, as well as the unexpected and inconclusive nature of the female-only results, provided the rationale for further investigation in a second study.

## Study 2

Given the uncertainty surrounding the results of study 1, we chose to conduct a partial conceptual replication that further examined the effects of supernatural and religious institutional primes on attitudes towards outgroup members only. Given that the differences observed in study 1 manifested almost entirely in terms of attitudes towards outgroup members (i.e., outgroup derogation), we chose to focus on outgroup attitudes in order to harness the increased power of a simplified 3 × 1 between-subjects design. As such, study 2 investigated only the effect of a single independent variable—prime content—on outgroup attitudes. As a consequence of this, we only tested hypotheses H1a and part of H2 in study 2.

The results of study 1 provided only very limited support for different effects of God and religion primes in the full, mixed-gender sample. While the results are not consistent with those observed by Preston and Ritter [45], subsequent analysis provided some evidence for an unanticipated gender effect, with the predicted deleterious effects of religious institutional (vs. supernatural primes) on outgroup attitudes being more pronounced in females. The purpose of study 2 was therefore two-fold. Firstly, we intended to re-examine the previous null effect of God and religion primes in a second mixed-gender sample while addressing the methodological concerns outlined in the study 1 discussion. Second, we intended to re-examine the moderating effect of gender on the prime-attitude relationship, in order to assess whether this surprising (and only partially supported) effect would replicate in a second sample.

### Method

#### Participants

As in study 1, participants in study 2 (N = 119) were undergraduate students from Nanyang Technological University in Singapore, who participated in return for partial course credit. Data from 15 participants were excluded from analysis as they indicated some suspicion of the experimental hypotheses (see the design and procedure section). In the remaining sample of 104 participants, 54.62% were female and 76.47% were ethnically Chinese. The percentages of participants who described themselves as ethnically Malay, ethnically Indian, or of another ethnicity, were 5.04%, 3.36%, and 2.52%, respectively. As before, all ethnic categorizations were made by the participants themselves. The mean age of the study 2 participants was 20.62 years (*SD* = 1.62). The sample was heterogeneous in terms of religious belief and affiliation. 21.01% of the participants identified as either Buddhist or Taoist, while Christians/Catholics comprised 23.53%. Of the other participants, 5.04% identified as Muslim, 2.52% as Hindu, while the remainder of the sample identified as either Free Thinkers (32.77%) or “others” (2.52%). Approval for the study was granted by the Nanyang Technological University institutional review board, and informed consent was obtained in all cases.

#### Design and procedure

Study 2 was administered remotely using the web-based survey platform Qualtrics [56]. Participants who registered for the study through the university participant recruitment system were given a computer-generated link which directed them to the survey. Participants were informed that the study was an investigation of personality and cognitive ability, and one of the tasks that was ostensibly supposed to assess cognitive ability—a spot-the-difference task (see the materials section for more details)—was included only as a way of subtly exposing participants to the experimental priming materials. The online survey platform was programmed to randomly allocate participants to one of the three priming conditions, which resulted in them being required to complete a different version of the spot-the-difference task. Following this, participants were asked to complete an indirect measure of attitudes towards an outgroup member, which in study 2 took the form of a vignette describing a hypothetical individual, followed by a pair of global evaluative questions (see the materials section for more information). A suspicion check and an attention check were also incorporated into the design, in order to identify cases of hypothesis awareness or failure to pay adequate attention to experimental instructions. To make the study’s cover story more convincing, the cognitive reflection test (CRT; [57]) was also included as an ostensive measure of cognitive ability. At the end of the study all participants read a debrief form informing them of the true nature of the experiment, and were awarded their course credit via the university’s system.

#### Materials

**Fig 2:**
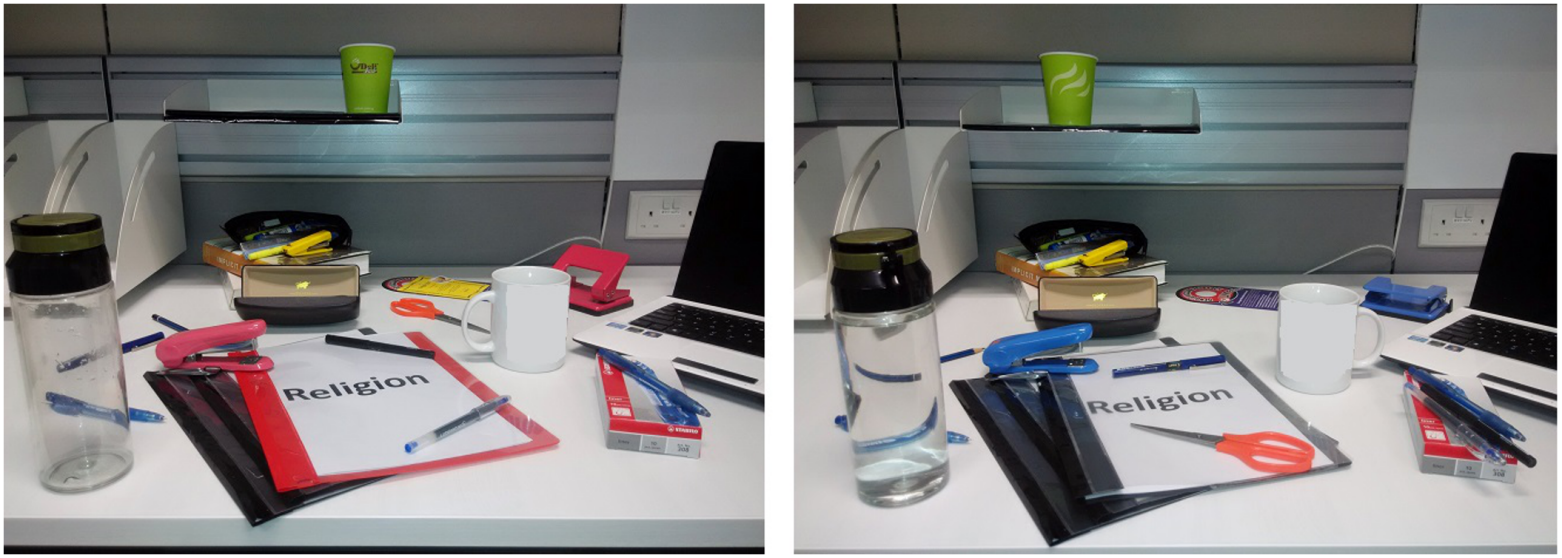
Image-pairs used in the "Spot the differences" task for the Religion priming condition.

God, Religion, and Neutral Primes: Participants were supraliminally primed with supernatural, religious institutional, or neutral imagery via the aforementioned spot-the-difference task. This task was designed in such a way that the words “God”, “Religion” or “Neutral” were displayed prominently, yet in a way that was subtle enough not to induce experimental demand. To accomplish this, two images containing the priming words were presented side by side on the computer screen, and participants were asked to spot as many differences as possible between the two pictures. In both images, the priming word was printed on the front cover page of a folder, meaning that the priming stimuli were effectively identical to those used during study 1. The images in the spot-the-difference task depicted the folders placed on a typical work desk, surrounded by various other paraphernalia. These distractor items were added to make the task appear more realistic and to enhance its face validity. In order to focus the attention of the participants on the priming word stimuli, the folder featuring the prime was placed in the center of the image, and the color of the folder was changed between the two images. In addition to this, most of the prominent differences between the two images in terms of arrangement of the unrelated items were focused around the folder containing the priming word stimuli. Fig 2 shows the image pairs used in the spot-the-difference task for the religion priming condition.

As a suspicion check, participants were asked to speculate on the experiment’s true purpose and phenomena of interest by choosing from the following options: “attitudes and prejudice”, “logic and reasoning”, “language fluency”, “perceptual ability”, “critical thinking”, and “memory”. Only 15 participants (13%) chose the first option. Of those participants who believed that the study investigated attitudes and prejudice, only one mentioned the religious primes or successfully identified the manipulation in their answers to an open-ended follow-up to the first suspicion check.

In order to ensure participants were paying adequate attention to the instructions given throughout the study, an attention check was performed in the form of a no-response question. Participants were instructed not to respond to the question, meaning that any answering of the questions indicated a lack of attention to instructions. Attention to the word primes in particular was also assessed towards the end of the experimental procedure, by asking participants to them to recall the word that was printed on the folders in the spot-the-difference task.

Measure of Attitudes towards Outgroup: Study 2 was designed to improve on study 1 both in terms of the attitude measure and the nature of the outgroup distinction. Firstly, in order to make the intergroup distinction more salient, study 2 departed from the subtle distinction of participants’ university affiliation in order to focus on the more divisive issue of citizenship. Immigrants are the targets of significant prejudice in Singapore [58] and a recent increase in anti-immigrant sentiment has been well-documented [59]. Adoption of this more important and obvious intergroup distinction addressed concerns over the choice of ingroup and outgroup in study 1. Additionally, as the results of study 1 suggested that the differential effects of God versus religion priming manifested predominantly (to the extent that they manifested at all) in differences in the evaluation of outgroup members by females, study 2 focused only on the effect of priming on outgroup evaluation. As mentioned earlier, this simplified 3 × 1 between-subjects design was adopted in order to maximize experimental power given limited access to additional research participants.

In order to measure attitudes towards immigrants, a vignette describing a foreign students’ characteristics and typical behaviors was presented to the participants. The name of this hypothetical person was typically Spanish-sounding (either “Adrian Garcia” or “Adriana Garcia”), something that would be extremely uncommon for a Singaporean, and the content of the vignette unambiguously indicated that the person in question was of foreign origin (“…has recently moved to Singapore from Spain”). To control for systematic error in attitude measurement resulting from the difference in gender of the participant and the gender of the person described in the vignette, the latter was gender-matched to the participants’ own gender, meaning that male participants made judgments about “Adrian Garcia” while female participants made judgments about “Adriana Garcia”. This allowed us to examine the effects of the different primes on attitudes towards outgroup members (foreigners) while controlling for possible confounds related to social status and gender. The vignettes presented to both male and female participants can be found in the S2 Appendix.

As an indirect measure of attitudes towards the immigrant, participants were asked to rate the person described on two dimensions: (a) likability (i.e., how much they thought they would like that person if they met them, and (b) willingness to spend time with them (i.e., how much they thought that they would like to spend time with that person). Responses for likability were measured on a six-point Likert scale anchored at 0 = *I wouldn't like them at all* and 5 = *I would like them a lot*. Similarly, responses for willingness to spend time were measured on a six-point Likert scale anchored at 0 = *I wouldn't like to spend time with them at all* and 5 = *I would very much like to spend time with them*.

### Results

The individual scores for likability and willingness to spend time were collapsed to give an overall measure of attitude towards the outgroup member. A one-way between-subjects ANOVA was conducted to compare the effect of priming condition on attitude towards outgroup in the God, religion, and neutral priming conditions. There was no significant effect of priming condition on attitude towards outgroup, *F*(2, 101) = .12, *p* = .88. These results were obtained after the exclusion of the fifteen participants who suspected that the study was actually measuring attitudes and prejudice, although the ANOVA results were similarly non-significant in the full sample. The results of study 2 therefore did not support either H1a or H2.

In order to examine whether these non-significant results were due to a lack of attention to study instructions, participants who failed the no-response question attention check (33 in total) were subsequently excluded from analysis and a one-way ANOVA was conducted to compare the effect of priming condition on attitude towards outgroup. There was no significant effect of priming condition on attitude (*p* = .89) even after excluding inattentive participants. Despite the high proportion of participants who failed the attention check, it is noteworthy that only 5% of those who failed the attention check also failed to correctly recall the word printed in the priming image folder. This accurate recollection of the prime words suggests that participants did in fact pay attention to the priming stimuli.

To examine whether the gender effects observed in study 1 replicated in the study 2 sample, a 2 × 3 ANOVA was conducted on attitude towards outgroup with gender and priming condition as the two independent variables. There was no significant effect of gender, *F* (1, 98) = .09, *p* = .76, or any two-way interaction between gender and priming condition, *F*(2,98) = .23, *p* = .80.

Finally, as there is evidence that the effect of religious priming is robust only for individuals who are sufficiently religious to begin with [60], we conducted the aforementioned ANOVA after exclusion of data from participants who identified as free thinkers. A 2 × 3 ANOVA on attitude towards outgroup with gender and priming condition as the two independent variables showed no significant effect of priming *F* (2, 59) = .54, *p* = .58, or gender, *F* (1, 59) = .04, *p* = .83. There was also no significant two-way interaction between gender and priming condition, *F*(2,59) = .19, *p* = .83.

### Discussion

Study 2 was conducted to test differential effects of God versus religion priming on attitudes towards an outgroup member, and was designed to improve on study 1 by using a more salient intergroup distinction and by employing a more global measure of attitudes towards outgroup members. Overall, the results showed no significant effect of priming on outgroup evaluation and thus replicated the null findings from study 1. This further supports the notion that God and religion primes do not have different effects on intergroup attitudes. In addition, the study 2 results indicated no significant effects of gender on the relationship between priming and outgroup attitudes. Since the unanticipated gender effect observed in study 1 was not replicated in a second sample, it seems prudent to conclude that the gender effect observed in study 1 is likely to have been a false positive.

## General Discussion

Previous research has indicated that religious institutional primes promote parochial altruism manifesting as enhanced prosociality towards ingroup members, while supernatural religious primes promote outgroup favoritism manifesting as enhanced prosociality towards members of ourgroups. While evidence for these divergent effects was recently obtained by Preston and Ritter [44] in the behavioral domains of charitable giving and cooperation in a laboratory-based social dilemma, the findings presented here do not seem consistent with their findings. In order to extend the findings of Preston and Ritter [44], we investigated the effects of God and religion primes on attitudes towards both ingroup and outgroup members in the specific domain of perceived competence (study 1), and also more general, global attitudes towards outgroup members (study 2). The central hypotheses were not supported in either study. In study 1, the critical interaction between prime and target group membership was found to be non-significant, while in study 2 the main effect of prime type was also found to be non-significant. These findings cast doubt on the divergent effects of God and religion primes, and raise the possibility of important cultural differences in the action of religious priming more generally.

Despite the null findings described above, our data did provide tentative evidence for religion-prime-induced parochial altruism in the female sub-sample of study 1, raising the possibility of an unexpected moderating effect of gender. Participants in the female sub-sample of study 1 who were exposed to a religion prime evaluated outgroup members significantly more negatively than ingroup members. However, it is also important to note that outgroup essay evaluations by females in study 1 were only marginally more negative than evaluations by females exposed to the neutral prime. In the presence of a true effect, one would expect both of these comparisons to yield significant effects. Furthermore, this surprising finding result did not replicate in a second sample. In study 2, females exposed to a religion prime did not evaluate outgroup members significantly more negatively than those exposed to a neutral control prime. This failure to replicate casts doubt on the veracity of the gender effect observed in study 1, and is more consistent with the overall tendency toward null priming effects in the present research.

To summarize, the two studies reported here provide little evidence of differing effects of God and religious primes specifically on intergroup attitudes. While this is to some extent surprising, the lack of evidence for more general religious priming effects is even more unexpected given the body of literature reporting such effects. In their recent meta-analysis of studies examining religious priming and prosociality, Shariff, Willard, Andersen, and Norenzayan [61] documented the relatively robust effects of different types of religious primes across 92 studies and reported a moderately sized average effect of *g* = .40 (although for a recent critique of this analysis, see [62]). In the subsequent sections we discuss possible explanations for both the former and the latter, as well as the possible (although somewhat unlikely) gender-moderated priming effect documented in study 1. Finally, we also discuss the implications of these findings for future work in the area of religious priming.

### Overt versus Covert Measurement of Dependent Variables

One possible explanation for the lack of observed effects relates to our adoption of attitudinal dependent variables. One issue with research on intergroup attitudes is tendency for individuals to respond in a socially desirable fashion, especially if they hold views which are considered politically incorrect by the majority. As such, negative attitudes towards minorities are often underestimated by more traditional, overt attitudinal measures [63], and discrepancies are often found between implicit and explicit measures of racism [64] and other forms of prejudice [65]. In addition to the obvious benefits of examining the effects of religious primes on actual behavior, one of the reasons that researchers frequently utilize behavioral measures is that the demand characteristics of these paradigms are relatively mild. Compared to a traditional overt attitudinal measure in which the participant is explicitly asked to indicate how much they like or dislike a certain social group, behavioral measures are often more subtle, involving the selective allocation of resources in economic games (e.g. [23]) where the group membership of the target is manipulated without them ever being explicitly referred to as ingroup or outgroup members. While people may be reluctant to endorse prejudicial attitudes because of concerns over the social desirability of their responses, prosocial actions can be framed more ambiguously (as in the prisoner's dilemma), meaning that the participants’ may be willing to willing to favor ingroup members over outgroup members when it is not immediately apparent that their actions reflect this tendency.

Although such an explanation of our null findings for is possible, it seems unlikely for several reasons. Firstly, significant effects of religious primes on self-reported attitudes towards both ingroup and outgroup members have previously been documented in several studies [27, 28]. Secondly, Shariff and colleagues [61] reported that the average effect size reported in religious priming studies that adopted self-report measures of religious prosociality were significantly larger than those adopting behavioral measures, a finding that casts doubt on the notion that behavioral measures may be more malleable and hence better able to detect the effects of religious primes. Thirdly, the attitudinal measures adopted in the present studies were not really overt, in that target group membership was manipulated in a way that was not immediately apparent to the participants. While responses to the funneled debrief questions in both study 1 and study 2 indicated that a small minority of participants (7% in study 1, 13% in study 2) correctly surmised that they were being asked to indicate their attitudes towards members of certain social groups, these cases were highly infrequent. Given the semi-implicit nature of our attitude measures and the well-documented susceptibility of even explicit attitude measures to priming effects, it seems implausible that the operationalization of the dependent variables could explain our null effects in both study 1 and study 2.

### Priming Methods

Another possible explanation of our results is that our priming methods were ineffective. The present studies used supraliminal priming methods (i.e., displaying a folder with the prime word written on its cover) in order to manipulate religious cognition. Given that all priming methods are necessarily covert [66], there is a possibility that our manipulation may have been too subtle, and participants may have either failed to pay adequate attention to the priming word stimuli, or failed to process their meaning in a significant way. Nonetheless, there are several reasons to believe that our priming methods were both appropriate and effective with respect to commanding the participants’ attention. In study 1 participants were left alone in small room for one minute with the priming materials displayed directly in front of them, which makes it seem unlikely that they would not have noticed the priming stimuli at all. Nonetheless, a reviewer of an earlier version of this manuscript raised the possibility that our participants may have noticed the priming materials without processing the words in a meaningful way. In order to address this concern, study 2 was designed in such a way that (a) attention to the priming materials was specifically requested, and (b) a manipulation check was included to confirm that participants had seen and processed the words featured on the covers of the folders. Firstly, completion of the spot-the-difference task is impossible without sustained attention on the items depicted in the two images. The images were structured in such a way that the folders were placed at the very center of each image, and displayed more prominently than any of the other items. Furthermore, most of the major differences between the two images of centered on the folder displaying the priming word, an intentional aspect of the task structure that should have drawn the participants’ attention to the priming stimuli. Responses to the manipulation check indicated that these measures were largely effective. When asked to recall the word displayed on the cover of the folder, only 14% of the participants in study 2 failed to recall the priming word. This indicates that the majority of participants were in fact paying attention to the priming stimuli.

It’s also important to note that robust priming effects have been elicited by far more subtle manipulations than the ones adopted in the present study. Significant religious priming effects have been documented using subliminal priming methods, [28, 26, 67, 68]. In such studies participants very rarely perceive or consciously process the meaning of the prime words, yet significant effects on attitudes and behavior have been observed. As such, it seems likely that more overt priming techniques would possess the strength or saliency required to be effective. This reasoning is supported by Shariff et al. [61] who reported an average effect size was *g* = .39 for studies employing supraliminal priming stimuli similar to those used in the present research. This figure compares favorably to the average effect size of *g* = .33 reported for studies using subliminal priming methods like the LDT.

### Sample Composition

It is worth noting that Shariff et al. [61] found the effects of religious priming to be consistent only for participants who are themselves religious. As mentioned by the authors, this indicates that religious priming may capitalize on culturally transmitted beliefs in the religious population rather than on just intuitive, low-level associations present in the general population. Still, it seems unlikely that sample composition could account entirely for the absence of a reliable priming effect across the two studies reported here, since the proportion of participants who reported no religious affiliation was only 25% and 33% in studies 1 and 2 respectively. As such, the majority of participants were religious and should in theory be susceptible to the effects of religious primes. Exclusion of participants who described themselves as free thinkers also had no effect on the main results of the analyses for either study 1 or study 2. Singapore is not a particularly non-religious nation, and the self-reported religiousness of the majority of our participants suggests that the religious composition of our samples cannot adequately explain our results.

### Cultural and Religious Differences

Another explanation for our results is a possible moderating effect of culture or religious affiliation. While Preston and Ritter’s [44] study was conducted at a university in the American Midwest with a predominantly Protestant and Catholic participant pool, our study had a more religiously diverse sample comprising significant numbers of Buddhists, Taoists, Protestants, Catholics, Muslims, and unaffiliated “free thinkers”. Furthermore, while Preston and Ritter [44] did not report the ethnicity or nationality of their participants, it is likely that majority were American citizens of White European descent. In contrast, the majority of our participants were ethnically Chinese Singapore citizens. It therefore possible that differences in conceptualizations of God or religion, either between religions or other cultural groupings (e.g., individualistic vs. collectivistic cultures, liberal vs. conservative cultures), could explain the differences between our results and those of Preston and Ritter [44]. Many researchers have noted religious priming has to be considered in terms of the cultural context and that the meaning of religious primes may change between cultures [69], given that they do not necessarily invoke the same associations among members of different cultural and religious groups.

This explanation is not, however, without its problems. Whereas the majority of research was until recently conducted in predominantly white, Christian samples from either Europe or North America [70], many researchers have recently extended this body of research to encompass ethnically and religiously diverse samples (e.g. [28, 60, 69]). While some of these studies have replicated and extended earlier findings, suggesting a degree of cross-cultural and pan-religious universality of these effects (e.g. [28]), others have failed to replicate classic findings from the recent literature (e.g. [69, 71]). Even those studies reporting similar effects have documented several differences in the ways these prime effects manifest. For example, Ramsay and colleagues [28] found that, while religious primes elicits prejudice towards non-value-violating outgroups in Asians as it does in Westerners, the specific groups that are targeted for prejudice varies between cultures.

Given that both cross-cultural similarities and differences in the effects of religious primes have been observed, it is unclear whether cultural differences can adequately explain the stark differences between these findings and the predominantly Euro-American literature. A convincing cultural explanation for the present null effects will require further experimentation and, most importantly, further theorizing on why religious primes may be ineffective in cultural settings that are otherwise fairly religious. Nonetheless, these results do underline the importance of conducting research in different cultures, and of refraining from making sweeping statements regarding the nature and function of religious cognition on the basis of evidence derived from monocultural samples.

### A Moderating Effect of Gender?

Despite the failure to replicate in study 2, a further discussion of the marginal gender effect observed in study 1 is warranted. The observation that religious primes enhance outgroup derogation only in females was unexpected, and deviates significantly from previous observations of prime-induced prejudice. To our knowledge, only one piece of religious priming research has reported a significant effect of gender [72] and further research by this same group failed to replicate this male-specific priming effect [73]. We are unaware of any previous demonstration of females being more receptive to the effects of religious priming than males. If indeed it is genuine, there are several possible explanations for this result. Firstly, it is well-documented that women tend to be more religious than men [55, 74]. This gender difference is robust, and researchers have observed significant gender differences in a range of indices of religious commitment, including those relating to spirituality and belief in the supernatural [75] and those relating to religious practice and community involvement [76]. It is possible that supernatural and institutional religious schemas may be more readily activated in females than males, a phenomenon that should in turn strengthen the cascading activation of related moral and ingroup protective cognitions in response to God and religion primes respectively.

However, the extant literature on religious priming does not indicate such differences, and few studies include gender as a moderator in their analyses. Given that this result did not replicate in a second sample, evidence for the effect is presently very limited, and it is prudent to conclude that the results of study 1 were spurious unless further instances of female-only religious priming effects are documented. Nonetheless, it is hoped that researchers in the field will be more inclined to test and discuss possible gender effects when investigating the effects of religious primes. Indeed, researchers in a related field have cautioned that “it may not be appropriate to generalize findings about the relationship between spirituality/religiosity and health from one form of spirituality/religiosity to another, across denominations, or to assume effects are uniform for men and women” ([77] p. 2848). Such cautionary words may also apply to other outcomes, and given the findings reported here, it is clear that possible gender effects should be more closely examined in future religious priming research.

### Replicating Previous Findings

Finally, it is important to consider whether the current findings imply that those obtained by Preston and Ritter were in fact false positives. Such a conclusion would at this moment be premature, considering that the current studies were envisaged as a conceptual (not direct) replication, and the methodological differences permit only limited comparison between the studies. While several authors have convincingly argued for the necessity of conceptual replications (e.g. [78]), a proper test of Preston and Ritter’s findings would require direct replication studies using the same behavioral materials. Even then, any difference in sample and experimental setting has the potential to produce a different set of findings.

More generally, a recent re-analysis of the Shariff et al. meta analysis [60] data has cast some doubt on the robustness of religious priming effects [61], with the authors suggesting that the field may suffer from considerable publication and experimenter bias that may have drastically inflated estimations of prime effectiveness. While the two studies reported here can only make a very limited contribution to this debate, the observation of null effects underscores the need for pre-registered direct and conceptual replications of widely cited studies documenting significant effects of religious priming.

## Conclusion

The present study provides little evidence for the different effects of supernatural and religious institutional primes on attitudinal judgments of ingroup and outgroup members. In study 1, individuals primed with the words “God”, “religion”, and a neutral control word evaluated both ingroup and outgroup members similarly, although a marginal tendency towards more negative evaluations of outgroup members by females exposed to religion primes was observed. In study 2, no significant differences in attitudes towards an outgroup member were observed between the God, religion, and neutral priming conditions. Furthermore, the gender effect observed in study 1 did not replicate in this second study. Most importantly of all, little evidence for the effectiveness of religious primes more generally was found in either study 1 or study 2. While it is possible that methodological issues could account for discrepancies between these findings and the wider literature, we suggest that cultural influence is a more likely candidate. Finally, we suggest that further evidence must be gathered if claims of the different effects of God and religion primes are to be substantiated.

## Supporting Information

S1 Appendix Essays used in the essay evaluation task in study 1. (PDF)

S2 Appendix Vignettes presented to male and females participants in study 2. (PDF)
