# Supplementary material for: A Puzzle Unsolved: Failure to Observe Different Effects of God and Religion Primes on Intergroup Attitudes
Source: PLoS One. 2016 Jan 26;11(1):e0147178. doi: 10.1371/journal.pone.0147178 (PMC4727913; doi:10.1371/journal.pone.0147178)
Supplement: S1 Appendix — (PDF) [file pone.0147178.s001.pdf]

S1 Appendix : Essays used in study 1

Ingroup Essay:

NANYANG TECHNOLOGICAL UNIVERSITY

2013 ESSAY CONTEST

Personal Details	
Name:	[redacted]
I.C. Number:	[redacted]
University:	Nanyang Technological University

Persuasive Writing Essay

Title: Why Should You Complain About Bad Service?

Essay:

The service sector is a very important economic activity in Singapore. Services include hotels, restaurants, schools, stores, etc. When we pay for a service, even if they are not selling us an object, they still must satisfy our needs. When we do not receive what we expect, we are not satisfied and we are paying for something that is not worth it. My purpose in this short essay is to explain the benefits of complaining so we can have enough reasons to act against bad or poor service.

When bad service is given and no one complains about it, people in the enterprise think they are doing a good job and they do not realize they are losing clients. People prefer just to walk away rather than talking. The consequence is that this enterprise will keep giving the same bad service. This is the first benefit of complaining: We will let them know what are they doing wrong and we will give them the chance to improve. Consequently, they will take actions to satisfy not only you who complained, but also the rest of the clients who did not dare or bother to complain and had the same need as you.

Another benefit of complaining is that the bad concept that people had about certain enterprises with bad service will change. The enterprise's reputation will improve and they will start offering service with quality thanks to previous complaints. If the service sector improves in quality, the Singaporean economic system will also improve.

The third benefit of complaining about bad service is that people who work in this sector will need to be more prepared and more open to hear their clients. If we have the habit of complaining when something does not satisfy us, enterprises will give us the respect we deserve as clients and the service we are looking for. They will understand they need to be nice and to improve. For such things, they need to have good job training and interest in what they do or work on.

Since the service sector is one of the most important ones in Singapore, it must be very well organized and it must always satisfy the needs of clients. Clients are not only Singaporeans but also tourists who come every year, such as those who visit for Formula One. All of them are also victims of our bad services. If we are paying for a service, we do not need to endure bad moods or receive something we were not looking for. We must complain. This way, we will give people the opportunity to change and we will improve not only as employers but also as persons and Singaporeans. This can help boost the image of Singapore around the world.

Declaration

I declare that the above essay is entirely my own work, and that I have read and understand the contest's guidelines on plagiarism.

Name:	[redacted]	Signature:	[redacted]	Date:	14/08/13

Outgroup Essay:

NUS

National University of Singapore

2013 ESSAY CONTEST

Personal Details

**Name:**	[redacted]
**I.C. Number:**	[redacted]
**University:**	National University of Singapore

Persuasive Writing Essay

Title: Why Should You Complain About Bad Service?

Essay:

The service sector is a very important economic activity in Singapore. Services include hotels, restaurants, schools, stores, etc. When we pay for a service, even if they are not selling us an object, they still must satisfy our needs. When we do not receive what we expect, we are not satisfied and we are paying for something that is not worth it. My purpose in this short essay is to explain the benefits of complaining so we can have enough reasons to act against bad or poor service.

When bad service is given and no one complains about it, people in the enterprise think they are doing a good job and they do not realize they are losing clients. People prefer just to walk away rather than talking. The consequence is that this enterprise will keep giving the same bad service. This is the first benefit of complaining: We will let them know what are they doing wrong and we will give them the chance to improve. Consequently, they will take actions to satisfy not only you who complained, but also the rest of the clients who did not dare or bother to complain and had the same need as you.

Another benefit of complaining is that the bad concept that people had about certain enterprises with bad service will change. The enterprise's reputation will improve and they will start offering service with quality thanks to previous complaints. If the service sector improves in quality, the Singaporean economic system will also improve.

The third benefit of complaining about bad service is that people who work in this sector will need to be more prepared and more open to hear their clients. If we have the habit of complaining when something does not satisfy us, enterprises will give us the respect we deserve as clients and the service we are looking for. They will understand they need to be nice and to improve. For such things, they need to have good job training and interest in what they do or work on.

Since the service sector is one of the most important ones in Singapore, it must be very well organized and it must always satisfy the needs of clients. Clients are not only Singaporeans but also tourists who come every year, such as those who visit for Formula One. All of them are also victims of our bad services. If we are paying for a service, we do not need to endure bad moods or receive something we were not looking for. We must complain. This way, we will give people the opportunity to change and we will improve not only as employers but also as persons and Singaporeans. This can help boost the image of Singapore around the world.

Declaration

I declare that the above essay is entirely my own work, and that I have read and understand the contest's guidelines on plagiarism.

**Name:**	[redacted]	**Signature:**	[redacted]	**Date:**	14/08/13
