# Supplementary material for: A Puzzle Unsolved: Failure to Observe Different Effects of God and Religion Primes on Intergroup Attitudes
Source: PLoS One. 2016 Jan 26;11(1):e0147178. doi: 10.1371/journal.pone.0147178 (PMC4727913; doi:10.1371/journal.pone.0147178)
Supplement: S2 Appendix — (PDF) [file pone.0147178.s002.pdf]

S2 Appendix: The vignettes presented to male and female participants

Vignette Presented to Male Participants

Adrian Garcia has recently moved to Singapore from Spain. He was awarded a scholarship by the Singapore Ministry of Education to pursue his undergraduate studies here. As a person he is very chirpy, and always strikes up a conversion with any stranger whenever there is an opportunity. In his free time, Adrian is on constant lookout for any sales event - be it at the mall or an on-line store, and tries to be the first to grab the best deals that are on offer. During weekends, he goes to play basketball with some of his male friends, a sport he has been playing since he was young. Whenever he is out with his friends, he always makes sure he has his say in deciding what activities to do, where to go or even where to dine.

Vignette Presented to Female Participants

Adriana Garcia has recently moved to Singapore from Spain. She was awarded a scholarship by the Singapore Ministry of Education to pursue her undergraduate studies here. As a person she is very chirpy, and always strikes up a conversion with any stranger whenever there is an opportunity. In her free time, Adriana is on constant lookout for any sales event - be it at the mall or an on-line store, and tries to be the first to grab the best deals that are on offer. During weekends, she goes to play basketball with some of her male friends, a sport she has been playing since young. Whenever she is out with her friends, she always makes sure she has her say in deciding what activities to do, where to go or even where to dine.
